# FLNA Expression in Cancer-Associated Fibroblasts Is Associated with Immunosuppression and Tumor Progression in Colorectal Cancer

**DOI:** 10.7150/jca.134223

**Published:** 2026-10-16

**Authors:** Qianze Yao, Ningbo Liu, Shilong Li, Bang Liu, Guozheng Ding, Hengbin Zhao, Weihua Fu

**Affiliations:** 1 Department of General Surgery, Tianjin Medical University General Hospital, Tianjin 300000, China.; 2 Department of Gastrointestinal Surgery II, Handan First Hospital, Handan 056000, China.

**Keywords:** FLNA, colorectal cancer, cancer-associated fibroblasts, tumor microenvironment, immunosuppressive microenvironment, prognosis

## Abstract

**Background:**

The role of Filamin A (FLNA) in colorectal cancer (CRC), particularly within the stromal compartment, remains poorly defined. This study investigates its expression, function, and clinical relevance in CRC.

**Methods:**

We integrated transcriptomic data from the GSE39582 training cohort and TCGA/GSE75316 validation cohorts. Analyses included functional enrichment, tumor microenvironment (TME) assessment (ESTIMATE, TIDE), immune correlation, and survival analysis. FLNA's cellular expression was delineated using single-cell RNA-seq data from 27,414 cells.

**Results:**

FLNA was upregulated in advanced-stage CRC and exhibited diagnostic potential in microsatellite instability (MSI) samples. Pathway analysis revealed a significant enrichment of FLNA in biological processes implicated in cell adhesion and remodeling of the extracellular matrix. High FLNA expression correlated with an immunosuppressive microenvironment, characterized by elevated stromal/immune scores, increased cancer-associated fibroblast (CAF) and macrophage infiltration, and exclusion of CD8⁺ T cells. It also showed strong associations with multiple immune checkpoints. Single-cell transcriptomics confirmed specific, high FLNA expression in CAFs, fibroblasts, and endothelial cells, where it was associated with pro-tumorigenic functions, including actin cytoskeleton organization. Furthermore, FLNA emerged as an independent predictor of adverse outcomes and was independently associated with reduced overall survival (OS).

**Conclusion:**

In summary, our findings suggest that FLNA expression is associated with tumor progression and an immunosuppressive microenvironment in CRC, and that FLNA warrants further investigation as a potential prognostic biomarker and therapeutic target.

## 1. Introduction

Globally, colorectal cancer (CRC) ranks third in cancer prevalence and accounts for the second highest number of cancer-related deaths 1, 2. In 2022, there were over 1.9 million new cases of CRC and approximately 904,000 deaths globally 3. In countries undergoing socioeconomic transition—including those in Eastern Europe, Southeast Asia, and South America—CRC incidence is increasing rapidly, reflecting its growing significance as a marker of socioeconomic development 4-6. Despite progress in targeted therapies and immunotherapy, survival outcomes among CRC patients continue to exhibit considerable heterogeneity 7, 8. The prognosis for early-stage CRC is excellent, with a 90% 5-year survival rate achievable through surgery. However, for patients with advanced, non-resectable tumors, the therapeutic landscape is markedly limited 9-11. Therefore, a deeper understanding of the mechanisms driving CRC progression and metastasis is imperative to pave the way for more effective, targeted therapeutic approaches.

CRC development is profoundly driven by its unique tumor microenvironment (TME), a complex and dynamic milieu composed of malignant cells, diverse immune infiltrates, cancer-associated fibroblasts (CAFs), a remodeled extracellular matrix (ECM), and dysregulated vasculature 12, 13. This multifaceted compartment orchestrates key oncogenic processes—from initiation and local invasion to distant metastasis and treatment failure—largely through its control over immune cell recruitment and function, angiogenesis, and ECM restructuring 14, 15. A comprehensive understanding of these pathways not only clarifies clinical heterogeneity but also lays the critical groundwork for future therapeutic development.

Within the TME of CRC, CAFs represent a critical stromal cell population. They secrete growth factors, inflammatory ligands, and ECM proteins, orchestrating pathological ECM remodeling that facilitates tumor proliferation, therapy resistance, and immune evasion—thereby fostering a permissive niche for tumor growth and invasion 16, 17. Zhang *et al*. 18 reported that SEMA3C, secreted by CAFs in CRC, binds to the NRP2 receptor and activates the MAPK pathway, promoting hepatic metastasis. Deng *et al*. 19 demonstrated that MYL9, which is predominantly expressed by CAFs in CRC, correlates strongly with M2 macrophage infiltration and contributes to an immunosuppressive microenvironment. Moreover, MYL9 governs the production of CCL2 and TGF-β1 from CAFs, thereby shaping an immunosuppressive landscape and driving tumor progression. In summary, CAFs act as pivotal accomplices in driving CRC progression within the TME. A deeper understanding of CAF heterogeneity, functional roles, and dynamic crosstalk with cancer cells and other microenvironmental components is vital for accurate clinical outcome prediction and the design of effective targeted interventions for CRC. It is noteworthy that CAF functions are governed by intricate intrinsic molecular networks. Although Filamin A(FLNA) has recently been implicated in malignant progression through aberrant expression in various tumor stromal contexts, its specific role in CRC CAFs remains poorly defined.

FLNA, formally known as Filamin A and also referred to as ABP-280 or ABPX, is a protein-coding gene that has been increasingly implicated in the pathogenesis of various cancers 20, 21. However, its regulatory role in CRC remains controversial. Xu *et al*. 22 linked calpain-1 overexpression to unfavorable clinical outcomes and enhanced tumor aggressiveness in CRC, a phenomenon potentially mediated by the downregulation of FLNA. In a separate study, Xu *et al*. 23 further uncovered a role for FLNA in orchestrating three-dimensional genomic reorganization in metastatic CRC. Through *in vitro* functional assays, they confirmed that FLNA depletion markedly compromises the motility and invasion of CRC cells. Further supporting its pro-tumorigenic role, Cheng and colleagues 24 implicated FLNA in conferring chemoresistance in CRC, by driving epithelial-mesenchymal transition (EMT) and stimulating the SMAD2 pathway. In contrast, our study reveals that FLNA is predominantly expressed within stromal cells in CRC, with the highest levels detected in CAFs. We further elucidate how FLNA expression influences malignant biological behaviors in CRC. Guided by these results, we aim to identify novel therapeutic targets and assess the value of FLNA for predicting both immunotherapy outcomes and prognosis in CRC.

## 2. Methods

### 2.1. Data collection and analysis

This study integrated transcriptomic data from three independent cohorts of patients with CRC: the GSE39582 cohort (n = 566), the GSE75316 cohort (n = 59) from the Gene Expression Omnibus (GEO), and The Cancer Genome Atlas (TCGA) cohort (n = 633). Raw data from each source were subjected to quality control, alignment, and normalization using platform-specific annotation files. The GSE39582 cohort was designated for training. The TCGA and GSE75316 cohorts were then repurposed as independent validation sets. Due to the limited availability of microsatellite instability (MSI) status annotations in the TCGA cohort, the GSE75316 cohort was used for validation in MSI-related analyses.

### 2.2. Functional enrichment analysis

We performed functional annotation of genes strongly correlated with FLNA, along with signature genes from key cell clusters, using the Database for Annotation, Visualization and Integrated Discovery (DAVID, v6.8). Analysis parameters included the use of standard gene nomenclature for identifiers and the human reference as the background species. The annotated gene sets were screened for enrichment in Gene Ontology (GO) terms, encompassing biological process (BP), cellular component (CC), and molecular function (MF) categories. Additionally, Kyoto Encyclopedia of Genes and Genomes (KEGG) pathway enrichment analysis was performed to identify signaling pathways significantly associated with the input gene sets. The resulting top five enriched terms or pathways, ordered by increasing Benjamini-Hochberg adjusted *p*-value, are reported.

### 2.3. Immuno-functional analysis

To evaluate the diagnostic performance of FLNA for discriminating MSI from microsatellite stable (MSS) tumors, receiver operating characteristic (ROC) curve analysis was performed and the area under the curve (AUC) was calculated. To explore the correlation between FLNA expression and the TME, we employed the R package “ESTIMATE” to compute stromal, immune, ESTIMATE scores, and tumor purity in patients with CRC. The ESTIMATE (Estimation of STromal and Immune cells in MAlignant Tumor tissues using Expression data) algorithm infers the fraction of stromal and immune cells in tumor samples based on gene expression signatures. The stromal score reflects the presence of stromal cells, the immune score captures the infiltration of immune cells, and the ESTIMATE score combines both to represent overall non-tumor cellularity. Tumor purity is derived as the complement of the ESTIMATE score, with lower purity indicating greater infiltration of non-tumor components. To quantify pathway activity in each CRC sample, we applied the “GSVA” package using its default parameters. The immune-related gene sets utilized in this analysis were retrieved from the GSEA repository (https://www.gsea-msigdb.org/gsea/index.jsp). The R package pheatmap was employed for the graphical representation of enrichment results in the form of heatmaps. Associations between FLNA expression and immune processes, as well as individual immune checkpoint genes, were evaluated using Pearson correlation analysis. To assess the potential for tumor immune escape, we applied the TIDE computational framework developed by Peng *et al*. 25, 26 through its online platform (http://tide.dfci.harvard.edu) to examine relationships between FLNA expression and TIDE scores, immunotherapy response, and tumor rejection signatures. Correlations between FLNA expression and abundances of specific cell types within the CRC TME were visualized using TIMER2 (http://timer.cistrome.org). Additionally, Pearson correlation was used to analyze FLNA associations with activation markers of CAFs as well as commonly secreted factors including chemokines, cytokines, and growth factors.

### 2.4. Single-cell sequencing

Single-cell transcriptomic data from 27,414 cells—isolated from tumor core, tumor-normal border, and normal mucosal tissues of six colorectal cancer patients—were obtained from the GEO database (GSE144735). Utilizing Seurat (v5.2.1), key preprocessing steps were carried out, including the standard procedures of quality control, data normalization, and correction for batch effects. Dimensionality reduction was carried out via principal component analysis (PCA), enabling cell clustering and visualization with UMAP. Cell types were annotated according to classical marker genes.

### 2.5. Survival analysis

We evaluated the association between FLNA expression levels and survival outcomes in CRC patients. The analysis included two independent cohorts: the GSE39582 dataset (n = 562, after excluding 4 patients with missing data) served as the training cohort, and TCGA CRC cohort (n = 619, after excluding 14 patients) was used as the validation cohort. In the training cohort, the optimal cutoff for FLNA expression was determined using the surv_cutpoint function from the R survival package, which identified a value of 9.37, corresponding to the top 20.11% of patients. To enhance clinical applicability and ensure consistency, a standardized threshold of the top 20% was applied to define high FLNA expression in the validation cohort. Cox proportional hazards regression analysis was performed to assess the independent prognostic value of FLNA expression. To ensure the validity of the multivariate model, we computed variance inflation factors (VIFs) for all clinical variables to check for multicollinearity. Variables with VIF > 5 were considered to indicate significant collinearity and were excluded from the final multivariable model (Supplementary Table S1).

### 2.6. Statistical analysis

Expression data processing and statistical analyses were performed using R software (version 4.4.3), except for Cox proportional hazards regression analyses, which were conducted using IBM SPSS Statistics (version 26.0). Pearson's correlation coefficient was used to assess correlations between continuous variables. For GO and KEGG enrichment analyses, *p*-values were adjusted using the Benjamini-Hochberg method, and a false discovery rate (FDR) < 0.05 was considered statistically significant. For all other analyses, a *p*-value < 0.05 was considered statistically significant.

## 3. Results

### 3.1. Elevated FLNA expression in CRC with high T stage, lymph node metastasis, and advanced clinical stage

Patients with varying levels of FLNA expression exhibited distinct clinical and pathological profiles. Heterogeneous distributions of FLNA expression, T stage, N stage, and overall clinical stage were observed across both the GSE39582 and TCGA cohorts (Figure 1A, B). Comparative analyses across subgroups showed that in the GSE39582 dataset, FLNA expression was significantly increased in samples with higher T stages (*p* = 0.0263, Figure 1C), lymph node metastasis (*p* = 0.0014, Figure 1D), and advanced clinical stages (*p* = 0.0032, Figure 1F). These results were independently validated in the TCGA cohort, where FLNA expression was similarly associated with higher T stage (*p* = 0.0109, Figure 1G), lymph node metastasis (*p* = 0.0017, Figure 1H), and advanced clinical stage (*p* = 0.0390, Figure 1J). Overall, these findings suggest that FLNA expression is elevated in more aggressive forms of CRC.

### 3.2. FLNA is associated with CRC cell adhesion and extracellular matrix remodeling via the focal adhesion-cytoskeleton signaling axis

To gain functional insights into FLNA, we examined its co-expressed gene network (*R* > 0.5, *p* < 0.05) using Pearson correlation. GO and KEGG pathway enrichment analyses were carried out using this gene set. In the GSE39582 dataset, the BP most significantly associated with FLNA included cell adhesion, homophilic cell adhesion via plasma membrane adhesion molecules, and cell-matrix adhesion (Figure 2A). The most enriched CC was the collagen-containing extracellular matrix (Figure 2B), and the leading MF was extracellular matrix structural constituent (Figure 2C). The most relevant signaling pathways involved cytoskeleton regulation and focal adhesion in muscle cells (Figure 2D). We validated these findings by identifying comparable enrichment patterns across all categories (BP, CC, MF, and KEGG) in the TCGA dataset (Figures 2E-H). Collectively, these findings suggest that FLNA expression is associated with cell adhesion and extracellular matrix remodeling in CRC.

### 3.3. FLNA as a potential diagnostic biomarker for MSI in CRC

We evaluated the relationship between FLNA expression and microsatellite status (MS-status). FLNA expression was significantly elevated in MSI patients compared to MSS patients in both the GSE39582 and GSE75316 datasets (Figures 3A, C). The diagnostic performance of FLNA for identifying MSI was assessed using ROC curve analysis. As expected, the AUC was 60.8% (*p* = 0.0067) in the GSE39582 dataset (Figure 3B) and 75.6% (*p* = 0.0440) in the GSE75316 dataset (Figure 3D). These results indicate that FLNA is significantly overexpressed in MSI tumors and thus possesses diagnostic value for identifying this CRC subtype. Given that MSI CRC patients often respond better to immune checkpoint inhibitors, we further hypothesize that FLNA expression may be linked to immunomodulatory features of the TME.

### 3.4. Elevated FLNA expression is associated with stromal-immune microenvironment activation in CRC

Interactions between tumor cells and the TME dynamically regulate metastatic progression. We re-analyzed the GSE39582 dataset using the ESTIMATE algorithm to compute stromal, immune, and ESTIMATE scores, as well as tumor purity (Figure 4A-D). The results showed significantly increased stromal score (*p* < 2.22e-16), immune score (*p* = 1.3e-15), and ESTIMATE score (*p* < 2.22e-16), along with significantly decreased tumor purity (*p* < 2.22e-16) in the high-FLNA expression group (Figure 4A-D). A consistent trend was observed in the independent TCGA cohort. Thus, high FLNA expression is associated with a remodeled TME in CRC.

### 3.5. High FLNA expression is positively correlated with pan-immune cell activation and enrichment of antigen-driven inflammatory pathways

During immunogenic cell death (ICD), activation of lymphocytes—including natural killer (NK) cells, T cells, and B cells—and the release of chemokines and cytokines collectively lead to apoptotic or necroptotic cancer cell death. To examine the influence of FLNA expression on immune pathway activity and cytokine signatures, we evaluated the enrichment of immune-related processes using GSVA in both the GSE39582 and TCGA datasets. Pearson analysis revealed a broad positive association between FLNA expression and most immune functional terms (Figure 5A). This correlation profile was upheld in the independent TCGA cohort (Figure 5B). These findings suggest that FLNA expression is associated with diverse immune processes and antigen-driven inflammatory pathways in the CRC microenvironment.

### 3.6. FLNA positively correlates with inhibitory immune checkpoints and is associated with immune escape features, including T cell exhaustion and exclusion, in CRC

Correlational analysis was extended to established inhibitory immune checkpoints (e.g., PD-1, PDL-1, CD200R1, LAG-3, TIM-3, CTLA-4, TIGIT) in the GSE39582 and TCGA datasets to probe the role of FLNA in tumor immunotherapy (Figures 6A, E). FLNA expression showed significant positive correlations with these checkpoints, suggesting a role in suppressing anti-tumor immune responses in CRC. Utilizing the TIDE framework (http://tide.dfci.harvard.edu), we further evaluated the relationship between FLNA expression and tumor immune evasion potential. Elevated FLNA expression was associated with higher TIDE scores (*p* < 2.22e-16; Figure 6B), inferior response to immune checkpoint inhibitors (ICIs) (*p* < 2.22e-16; Figure 6C), and elevated exclusion scores (*p* < 2.22e-16; Figure 6D). These results were corroborated in the TCGA cohort (Figures 6F-H). Together, these findings suggest that high FLNA expression is associated with immune escape features, including T cell dysfunction and exclusion, and with a poorer predicted response to immunotherapy.

### 3.7. FLNA expression is associated with infiltration of CAFs and macrophages and reduced CD8⁺ T cell infiltration in an immunosuppressive TME

**To investigate the influence of FLNA on immune cell composition in the TME, we assessed its correlation with various cellular components using the TIMER2 database (EPIC algorithm). FLNA expression exhibited strong positive correlations with the infiltration levels of CAFs (*ρ* = 0.758, *p* = 1.57e-52), endothelial cells (*ρ* = 0.592, *p* = 2.01e-27), and macrophages (*ρ* = 0.519, *p* = 2.28e-20) (Figure 7A, B and F). In contrast, a significant negative correlation was observed with cytotoxic CD8⁺ T cell infiltration (*ρ* = -0.252, *p* = 2.42e-05) (Figure 7D).** Collectively, these data indicate that elevated FLNA expression is associated with an immunosuppressive TME characterized by increased CAF and macrophage infiltration and reduced cytotoxic CD8⁺ T cell infiltration.

### 3.8. FLNA is associated with TME remodeling through CAF activation and secretory function in CRC

We observed a strong correlation between FLNA and CAFs. FLNA showed strong positive correlations with established CAF activation markers in CRC, including ACTA2, THY1, FAP, S100A4, and PDPN (Figure 8A). In addition, significant positive correlations were observed between FLNA and eight CAF-derived chemokines, cytokines, and growth factors — CCL2, CXCL12, IL6, HGF, TGFB1, TGFB3, VEGFB, and VEGFC — which are well-documented components of the CAF secretome 27-30 (Figure 8B, C). These data suggest that FLNA expression is associated with TME remodeling, CAF activation, and the CAF secretory profile.

### 3.9. Single-cell transcriptomic analysis identifies specific FLNA expression in stromal cells of CRC

Unsupervised clustering and t-SNE visualization identified 13 distinct cell subpopulations (Supplementary Figure S1). Cell types were annotated based on canonical marker genes (Figure 9C): cluster 10 was annotated as CAFs, clusters 4 and 5 as fibroblasts, and cluster 8 as endothelial cells (Figure 9A). FLNA expression was highest in CAFs (cluster 10), followed by fibroblasts (clusters 4 and 5) and endothelial cells (cluster 8) (Figure 9B). Consistent with our annotation, FLNA was previously identified as a marker of a specific CAF subpopulation in CRC 31.

### 3.10. FLNA expression is associated with pro-tumorigenic functions in stromal cells, including actin cytoskeleton organization and the "Proteoglycans in cancer" pathway

Comparison of BP and KEGG pathways across CAF, fibroblast, and endothelial clusters stratified by FLNA levels revealed convergent biological functions and pathway activation profiles in high-FLNA clusters (Figure 10A, C, E). Specifically, the BP of actin cytoskeleton organization was enhanced in CAF and endothelial clusters with high FLNA expression (Figure 10A, B, E, F), while the "Proteoglycans in cancer" pathway was markedly enriched in fibroblast clusters with elevated FLNA (Figure 10C, D). These results suggest that FLNA expression is associated with pro-tumorigenic functional programs in stromal cells.

### 3.11. FLNA serves as an independent predictor of overall survival (OS) in CRC

Kaplan-Meier analysis revealed that the median OS was significantly shorter in patients with high FLNA expression (2730 days) compared to those with low expression (4350 days) in the GSE39582 cohort (Figure 11A). This prognostic significance was confirmed in the TCGA cohort (Figure 11B). After excluding T and N classifications due to multicollinearity with stage (Supplementary Table S1), multivariable Cox regression analysis showed that FLNA expression remained an independent prognostic factor (Tables 1 and 2). These results indicate that FLNA is an independent predictor of survival in both the GSE39582 and TCGA datasets.

## 4. Discussion

CRC bears a substantial global burden, ranking among the foremost causes of illness and death within gastrointestinal malignancies. Its pathogenesis involves a multi-step carcinogenic process driven by complex genetic and epigenetic alterations. Effectiveness of clinical management depends critically on early detection; however, approximately 40% of patients are diagnosed with lymph node metastasis at initial presentation 32, 33. Those with advanced disease confront poor prognosis and restricted therapeutic options. Notably, although ICIs have improved outcomes in MSI CRC patients, primary and acquired resistance to immunotherapy remains a substantial clinical challenge. Against this backdrop, the discovery of novel biomarkers that can predict tumor progression, metastatic risk, and immunotherapy response is of crucial importance.

As a filamentous actin-binding protein, FLNA serves as a central scaffold for cytoskeletal organization and signal transduction 34, 35. It orchestrates a wide range of processes—from cell adhesion, migration, and mechanotransduction to the regulation of gene expression, proteolysis, and ion channel activity—through interactions with over 90 partner proteins 36-38. However, the role of FLNA expression and variation in CRC remains incompletely explored, particularly with respect to its integrated effects on tumor progression, patient survival, and immunomodulation. Our findings suggest that FLNA is associated with clinically aggressive features in CRC and may contribute to an immunosuppressive tumor microenvironment. Using multi-cohort clinical data and single-cell sequencing, this study provides evidence that FLNA expression in CAFs is linked to stromal remodeling and immune evasion, warranting further investigation as a potential therapeutic target.

Leveraging public mRNA-seq data (GEO and TCGA), we profiled FLNA expression variations across pathological stages in CRC. Our results indicate that FLNA is upregulated in cancers of higher malignancy, particularly those with higher T stages, lymph node metastasis, and advanced clinical stages, suggesting that FLNA expression is associated with tumor progression, invasiveness, and metastatic spread. Our GO/KEGG findings are consistent with the known involvement of FLNA in cell adhesion and extracellular matrix remodeling via focal adhesion-cytoskeleton signaling 39, 40.

In CRC, MSI tumors exhibit elevated tumor mutational burden and dense lymphocytic infiltration, features that typically predict heightened sensitivity to PD-1 blockade therapy 41-43. Paradoxically, while FLNA is robustly expressed in MSI CRC and demonstrates diagnostic value, it co-occurs with a marked immune-excluded phenotype. Specifically, elevated FLNA expression was linked to broad activation of immune pathways—consistent with typical MSI-associated immunogenicity—as evidenced by GSVA and ESTIMATE analyses. Yet, high FLNA levels also correlated strongly with multiple markers of T cell exhaustion, including PD-1, LAG-3, and TIM-3. Further TIDE analysis revealed that high FLNA expression was associated with an immunosuppressive TME state, characterized by increased immune escape potential, elevated exclusion scores, and a poorer predicted response to ICIs. Recent studies further support a role for FLNA in mediating immune evasion through regulation of PD-L1 expression and immune cell infiltration, thereby facilitating CRC progression 44. Collectively, these findings highlight an apparent immunological paradox in MSI CRC. While high FLNA expression was associated with broad activation of immune-related pathways, consistent with the inherently immunogenic phenotype of MSI tumors, it was also accompanied by higher TIDE scores, increased T-cell exclusion, and a less favorable predicted response to ICIs. These observations suggest that immune activation alone may be insufficient to elicit effective antitumor immunity in FLNA-high tumors.

A potential explanation is that ECM remodeling associated with high FLNA expression reshapes the stromal architecture to form a physical barrier that restricts T-cell trafficking and infiltration into the tumor parenchyma, thereby attenuating the antitumor immune response despite the immunogenic background of MSI tumors. In addition, given the close association between FLNA and CAF-related biological processes observed in our study, FLNA expression may be associated with increased secretion of CAF-derived cytokines and chemokines, which in turn may contribute to immune exclusion and suppression of T-cell function. Together, these possibilities may partially account for the discordance between enhanced immune-related signaling and the unfavorable immunotherapy-related features observed in FLNA-high tumors.

Although these mechanistic links remain speculative, our findings provide a rationale for further experimental studies to determine whether FLNA actively orchestrates stromal remodeling and immune evasion in MSI CRC, and whether targeting FLNA-mediated CAF remodeling could improve the efficacy of immune checkpoint blockade.

Additionally, analysis via the TIMER2 database (EPIC algorithm) confirmed significant positive correlations between FLNA expression and infiltration of CAFs (*ρ* = 0.758, *p* = 1.57e-52), endothelial cells (*ρ* = 0.592, *p* = 2.01e-27), and macrophages (*ρ* = 0.519, *p* = 2.28e-20), along with a notable negative correlation with CD8⁺ T-cell infiltration (*ρ* = -0.252, *p* = 2.42e-05). These findings further suggest that FLNA expression is associated with ECM remodeling and CAF activation, and with features consistent with physical exclusion and impaired recruitment of antitumor effector T cells, which may contribute to immune escape. Concurrently, strong positive correlations were observed between FLNA and established CAF activation markers (e.g., ACTA2, FAP) as well as protumorigenic secretory factors derived from CAFs—including CXCL12, CCL2, TGF-β, HGF, and VEGFC—suggesting that FLNA expression is associated with both CAF activation and a protumor secretory phenotype. Previous studies have shown that TGF-β and HGF promote invasion and migration in colon cancer 45, 46; that CAF-derived CCL2 activates STAT3 signaling in endothelial cells to stimulate angiogenesis 47; and that CAF-secreted CXCL12 recruits macrophages and induces M2 polarization, thereby modulating CRC cell function and cisplatin resistance 48. Moreover, VEGFC is widely recognized as a central inducer of lymphangiogenesis, facilitating lymph node metastasis. Tacconi *et al*. 49 further demonstrated that in primary CRC, the VEGFC/VEGFR3 axis reshapes the lymphatic endothelium and tumor-associated macrophages, fostering an immunosuppressive microenvironment that accelerates tumor progression. Our single-cell sequencing analysis corroborated the stromal-specific expression of FLNA, with highest expression observed in CAFs, followed by fibroblasts and endothelial cells. Moreover, CAF and endothelial clusters with high FLNA expression exhibited enhanced enrichment in the actin cytoskeleton organization pathway, suggesting that FLNA expression is associated with cytoskeletal reorganization and matrix-related signaling within stromal cells. In summary, FLNA is predominantly expressed by CAFs in CRC, and its expression is associated with an immunosuppressive TME characterized by CAF activation. On the one hand, high FLNA expression is associated with ECM remodeling that may physically exclude T-cell infiltration; on the other hand, it is associated with the secretion of immunosuppressive factors such as CXCL12, TGF-β, CCL2, and VEGFC, which may directly inhibit antitumor immunity and are associated with tumor proliferation, invasion, and migration. Together, these observations suggest that FLNA expression is associated with immune evasion and tumor progression in CRC, although the causal relationships remain to be established.

Although this work provides insights into the potential role of FLNA in the CRC TME, several limitations warrant consideration in future studies. Firstly, our findings are derived primarily from bioinformatic mining and correlational analyses of public databases including GEO and TCGA. Although the use of multiple independent cohorts strengthens the reliability of the results, the retrospective nature and potential selection biases of these databases may limit the generalizability of our conclusions. Secondly, while transcriptomic and computational approaches have suggested potential mechanisms underlying the observed associations between FLNA and CAFs or the TME, these insights lack direct functional validation through *in vitro* and *in vivo* experiments. Future studies will therefore focus on experimental validation using cellular and animal models to further elucidate the biological functions and potential mechanisms involving FLNA in CRC.

## 5. Conclusion

In summary, our findings suggest that FLNA expression is associated with tumor progression and an immunosuppressive tumor microenvironment in CRC, highlighting its potential involvement in stromal-immune crosstalk. Given its consistent association with poor prognosis, immune exclusion, and CAF activation across multiple independent cohorts, FLNA warrants further investigation as a potential biomarker and therapeutic target. Future experimental studies are needed to clarify the functional role of FLNA in CAFs and to determine whether targeting FLNA or its downstream pathways can remodel the TME and improve therapeutic outcomes.

## Supplementary Material

Supplementary figure and table.

## Figures and Tables

**Figure 1:**
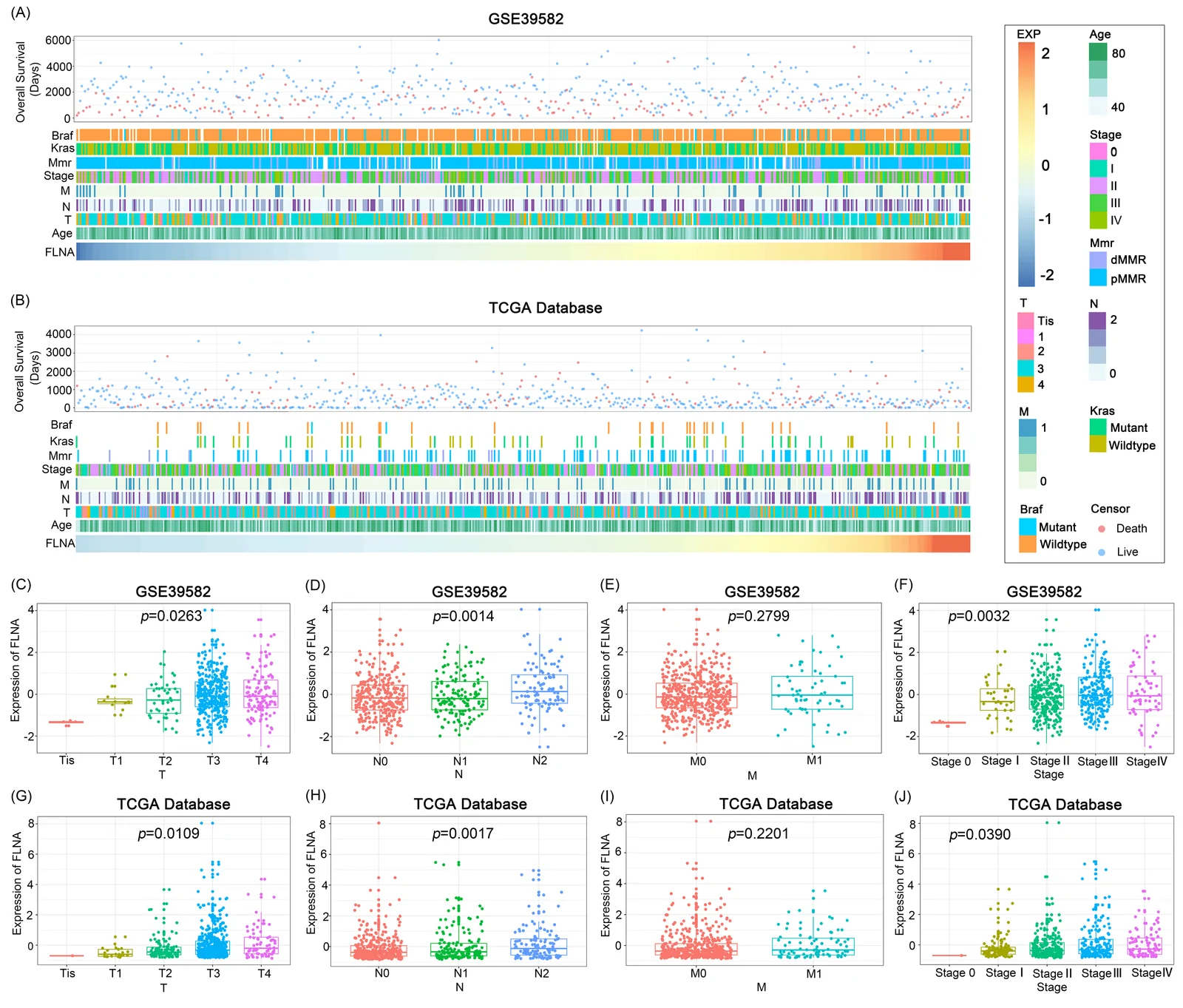
Association of FLNA with clinicopathological characteristics in colorectal cancer (CRC). (**A**) Profile of FLNA-associated clinicopathological features in CRC from the GSE39582 dataset. (**B**) Profile of FLNA-associated clinicopathological features in CRC from The Cancer Genome Atlas (TCGA) database. (**C** and** G**) FLNA expression was significantly elevated in CRC with higher T-stage disease in the GSE39582 and TCGA cohorts, respectively (one-way ANOVA). (**D** and** H**) FLNA expression was significantly elevated in CRC with lymph node metastasis (high N-stage) in both GSE39582 and TCGA databases (one-way ANOVA). (**E** and** I**) FLNA expression was increased in CRC with distant metastasis (M1 stage) in the GSE39582 and TCGA datasets; however, the difference was not statistically significant (unpaired t-test). (**F** and** J**) FLNA expression was significantly elevated in advanced-stage CRC within both the GSE39582 and TCGA cohorts (one-way ANOVA).

**Figure 2:**
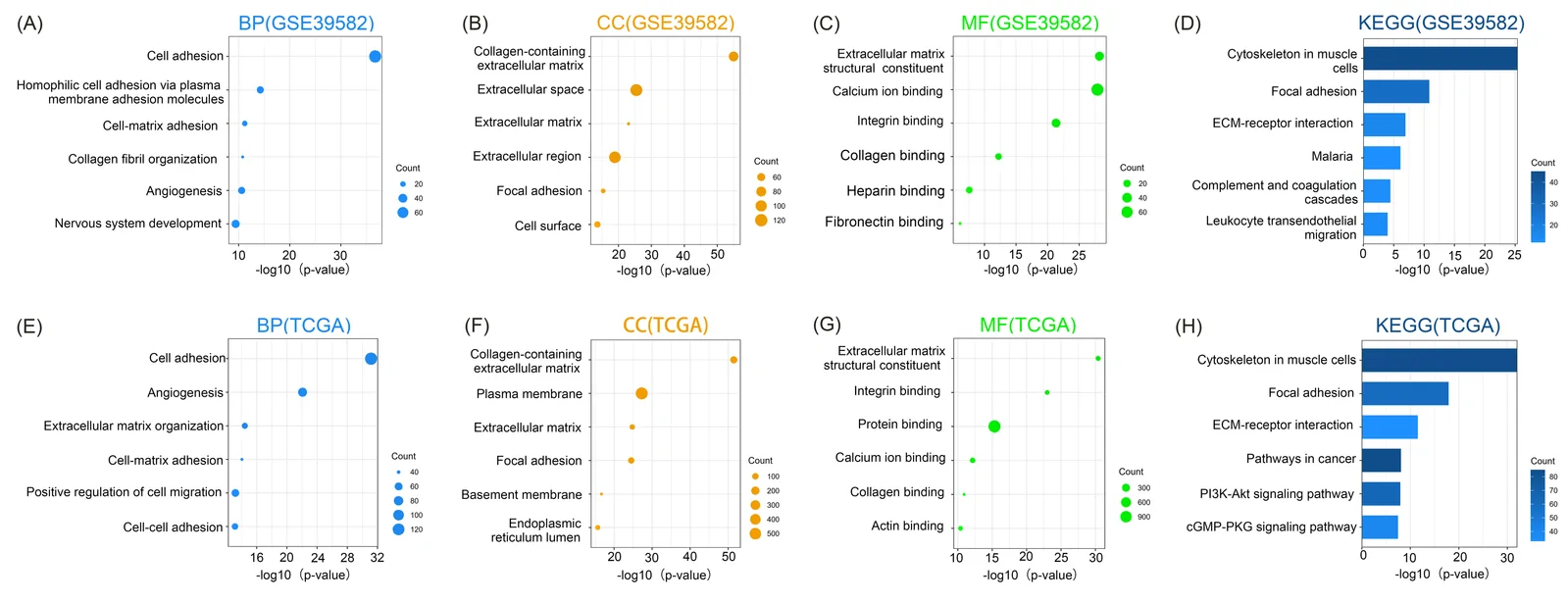
FLNA is closely associated with cell adhesion and extracellular matrix remodeling in colorectal cancer. (**A**-**C**) In the GSE39582 dataset, Gene Ontology (GO) enrichment analysis shows significant associations of FLNA with biological processes (BP), cellular components (CC), and molecular functions (MF). (**D**) Kyoto Encyclopedia of Genes and Genomes (KEGG) pathway analysis of FLNA in the GSE39582 dataset. (**E**-**G**) Similarly, in The Cancer Genome Atlas (TCGA) database, FLNA is significantly associated with BP, CC, and MF based on GO analysis. (**H**) KEGG pathway analysis of FLNA in the TCGA database.

**Figure 3:**
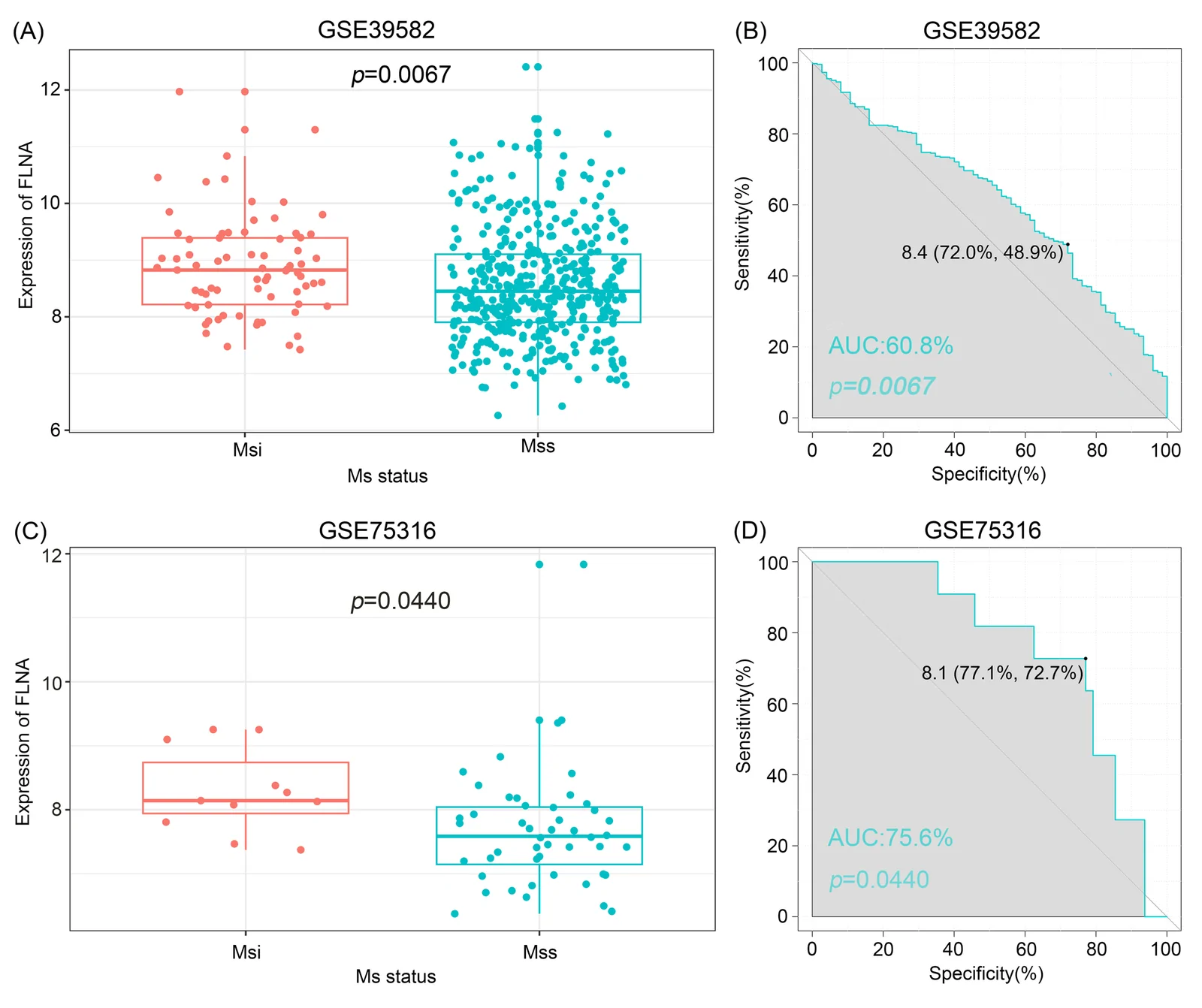
FLNA expression is elevated in microsatellite-instable (MSI) colorectal cancer (CRC). (**A** and** C**) FLNA expression is significantly higher in MSI CRC compared to microsatellite-stable (MSS) tumors in both the GSE39582 and GSE75316 datasets (unpaired t-test). (**B** and **D**) Receiver operating characteristic (ROC) curves demonstrate the diagnostic performance of FLNA expression in distinguishing MSI from MSS CRC in the GSE39582 and GSE75316 cohorts. AUC, area under the curve. MS-status, microsatellite status.

**Figure 4:**
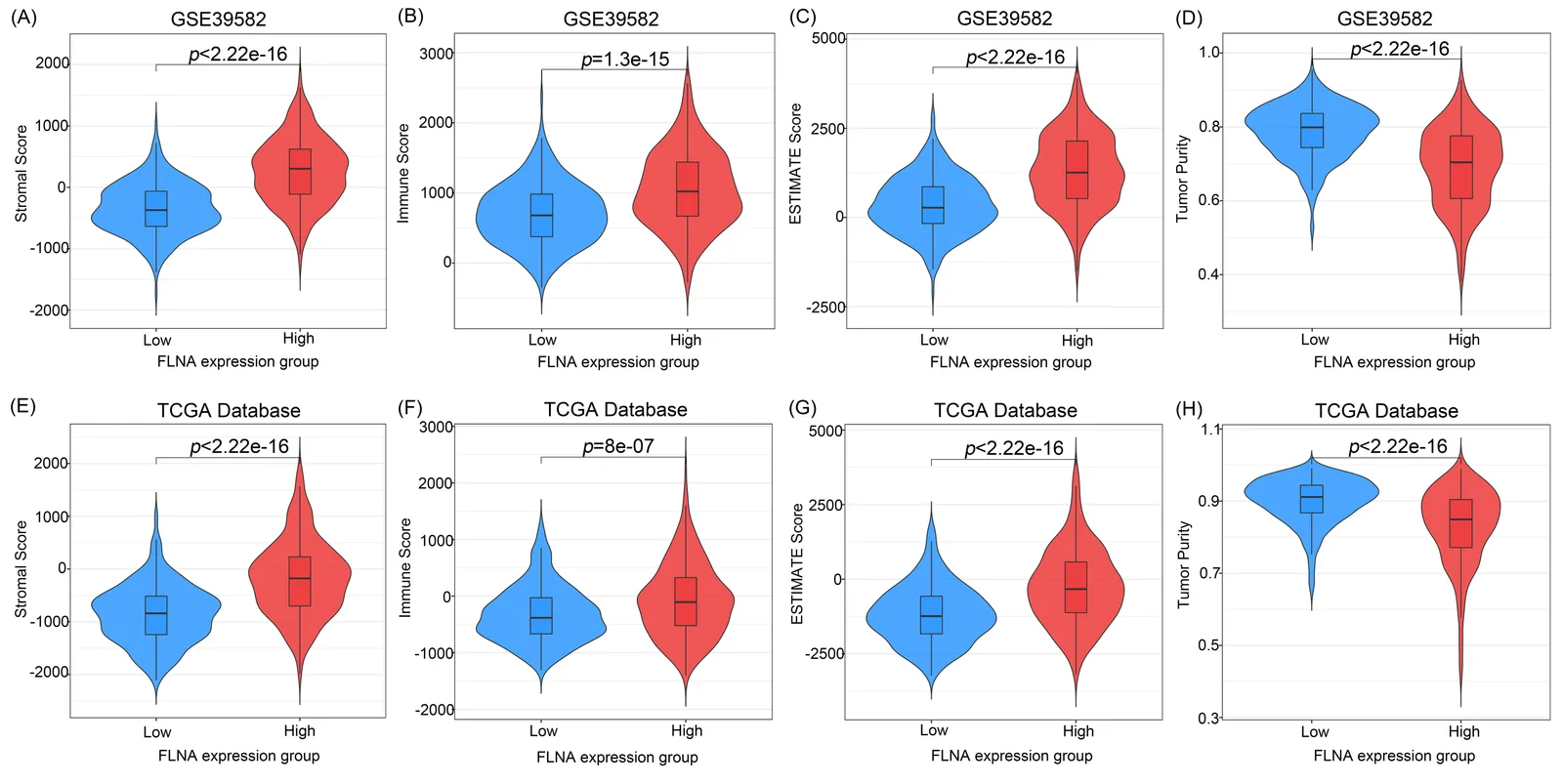
FLNA expression is associated with remodeling of the tumor microenvironment (TME) in colorectal cancer. (**A**-**D**) Re-analysis of the GSE39582 dataset using the ESTIMATE algorithm showed significantly higher stromal, immune, and ESTIMATE scores, along with lower tumor purity, in the high-FLNA group compared to the low-FLNA group (Wilcoxon rank-sum test). (**E**-**H**) Consistent results were observed in The Cancer Genome Atlas (TCGA) cohort, confirming that high FLNA expression is associated with increased stromal, immune, and ESTIMATE scores, and decreased tumor purity (Wilcoxon rank-sum test).

**Figure 5:**
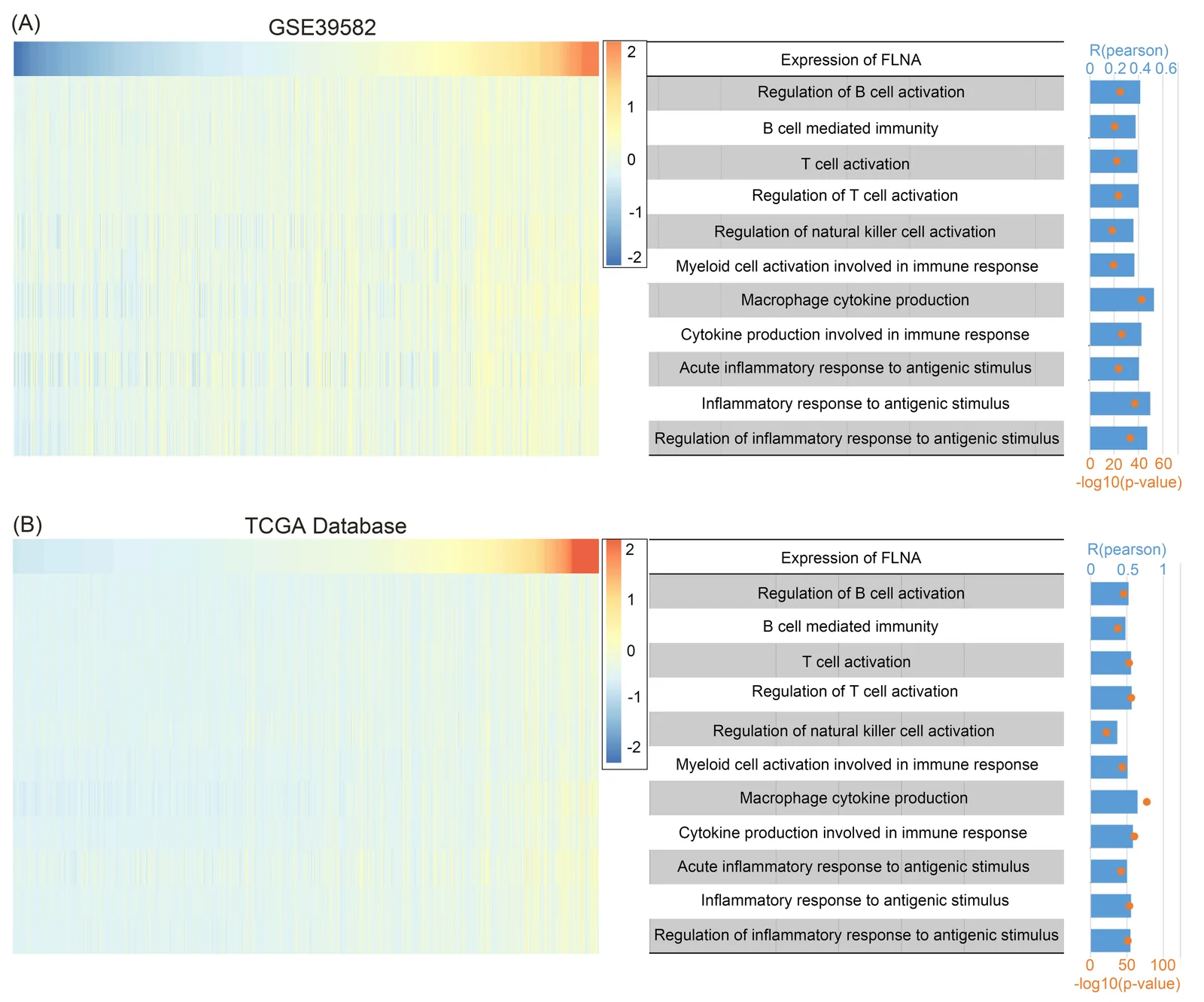
Correlation of FLNA Expression with Immune Function Enrichment Scores. (**A** and** B**) Heatmaps depict FLNA expression and immune function enrichment scores for each patient in the GSE39582 and The Cancer Genome Atlas (TCGA) databases. Samples were sorted from lowest to highest based on their FLNA expression. The corresponding bar and line plots summarize the statistical metrics (*R* and *p*-value) for the correlation.

**Figure 6:**
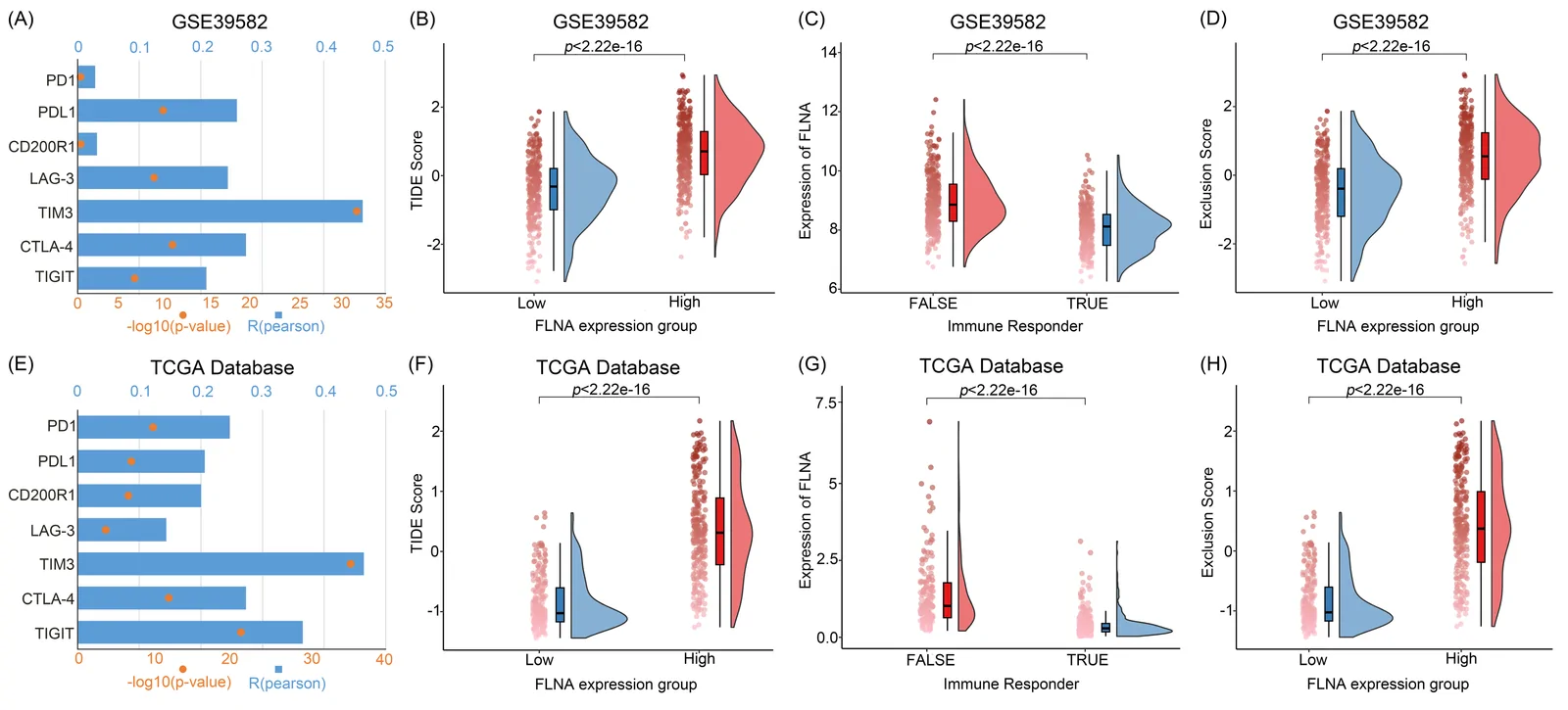
FLNA Expression Associates with Inhibitory Immune Checkpoints and Drives Immune Escape. (**A** and** E**) Analysis of the GSE39582 and The Cancer Genome Atlas (TCGA) databases shows that FLNA expression is significantly positively associated with multiple inhibitory immune checkpoint molecules. (**B**-**D**, **F**-**H**) TIDE analysis further confirms that high FLNA expression is correlated with increased immune escape potential (TIDE score), poorer predicted response to immunotherapy, and T cell exclusion. Significance was evaluated by Wilcoxon rank-sum test.

**Figure 7:**
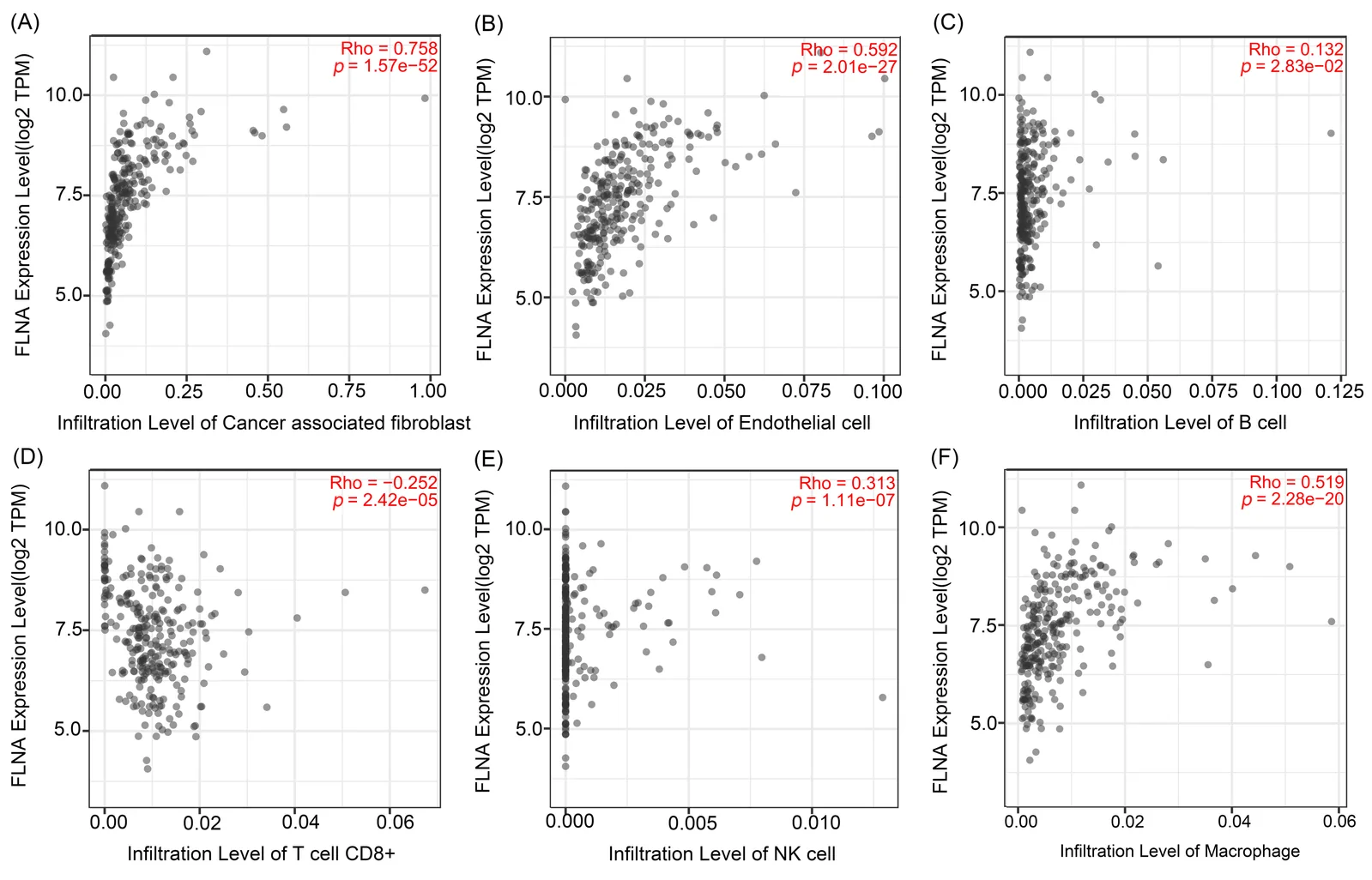
FLNA expression correlates with immune cell infiltration in the tumor microenvironment (TME). The EPIC algorithm (TIMER2 database) was applied to quantify associations between FLNA expression and immune cell infiltration levels.

**Figure 8:**
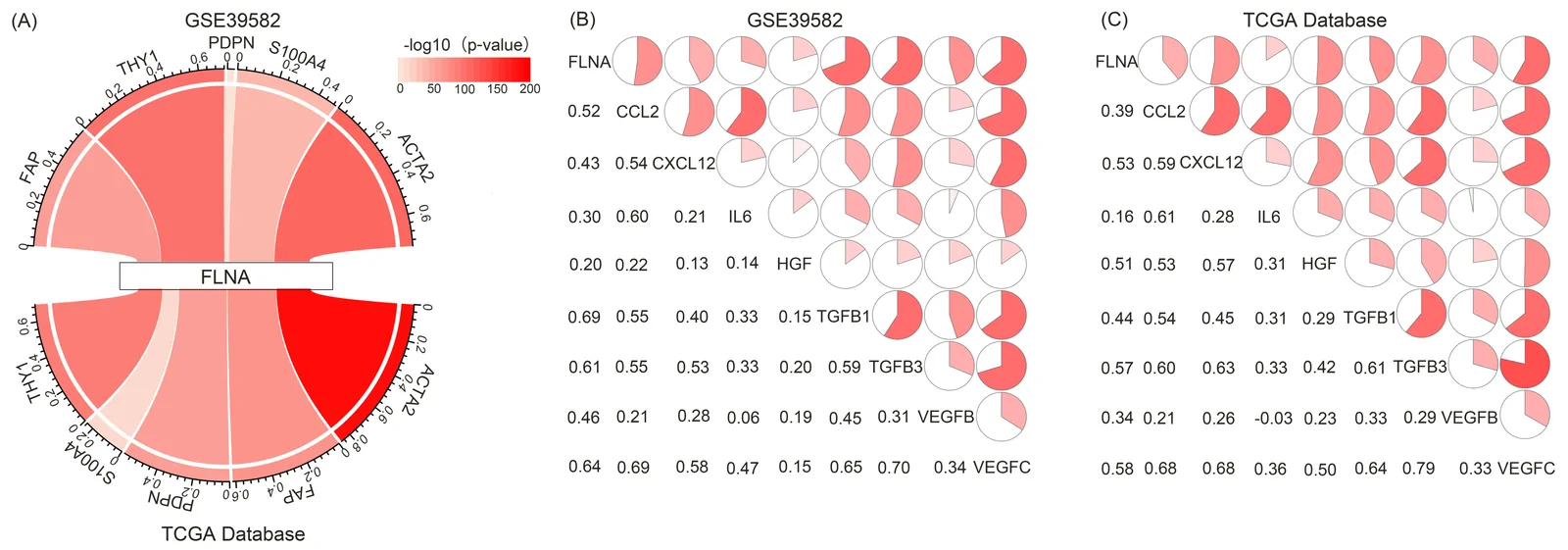
Association of FLNA expression with cancer-associated fibroblast (CAF) activation markers and secreted signaling molecules in GSE39582 and The Cancer Genome Atlas (TCGA) cohorts. (**A**) Pearson correlation between FLNA and established CAF activation markers. Band width corresponds to the absolute correlation coefficient (*R*), and band color indicates the *p*-value. (**B** and **C**) Correlation matrices between FLNA and various signaling molecules secreted by CAFs. Positive and negative correlations are denoted by red and green, respectively. Correlation coefficients are displayed numerically in the lower left quadrant and proportionally within pie charts. Pearson correlation analysis was used throughout.

**Figure 9:**
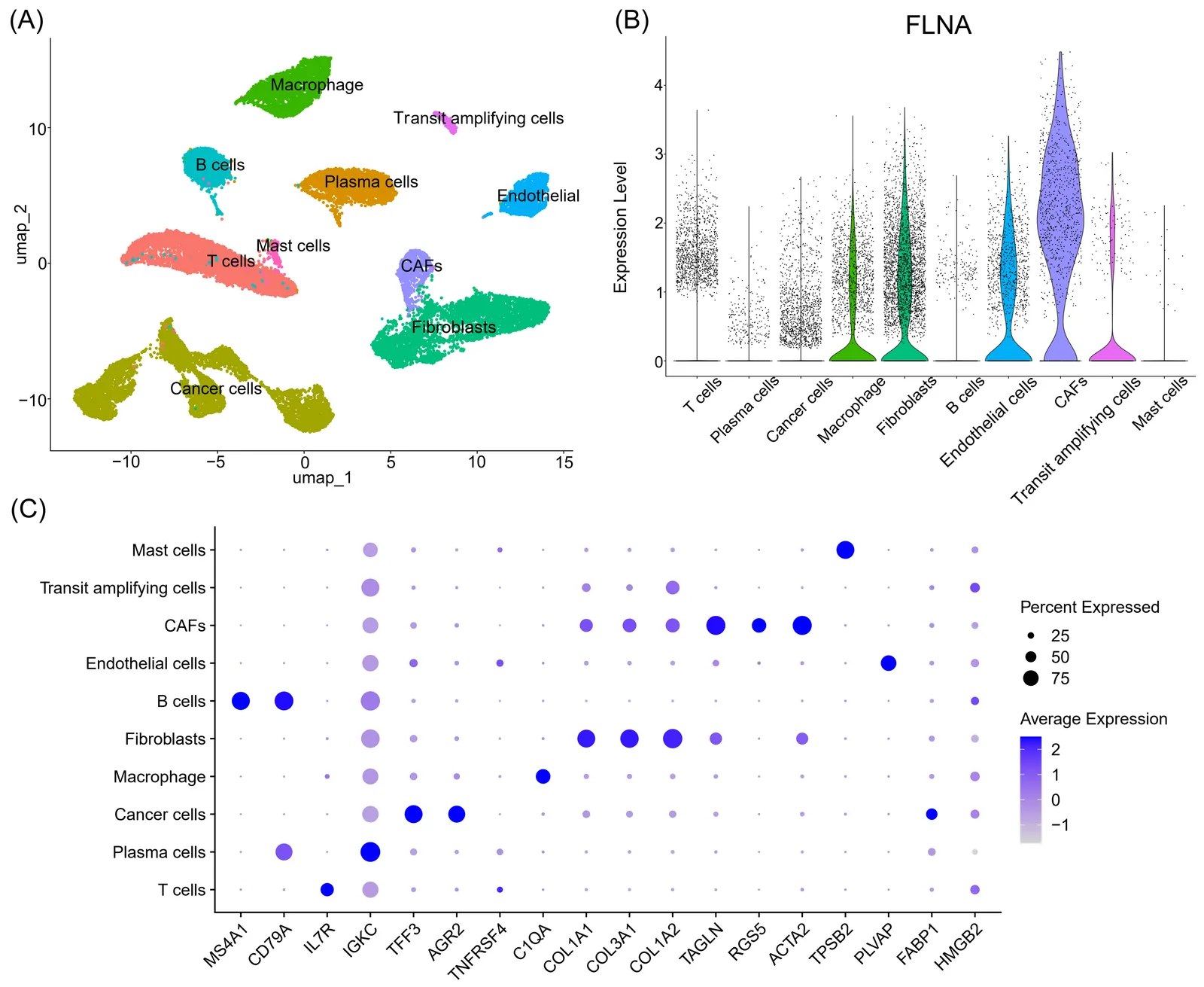
Expression of FLNA across cell types in the colorectal cancer (CRC) tumor microenvironment (TME). (**A**) UMAP plot showing cell clusters based on transcriptomic profiles from the GSE144735 dataset. (**B**) Distribution of FLNA expression levels across the identified cell clusters. (**C**) Cell type annotation performed using classical marker genes.

**Figure 10:**
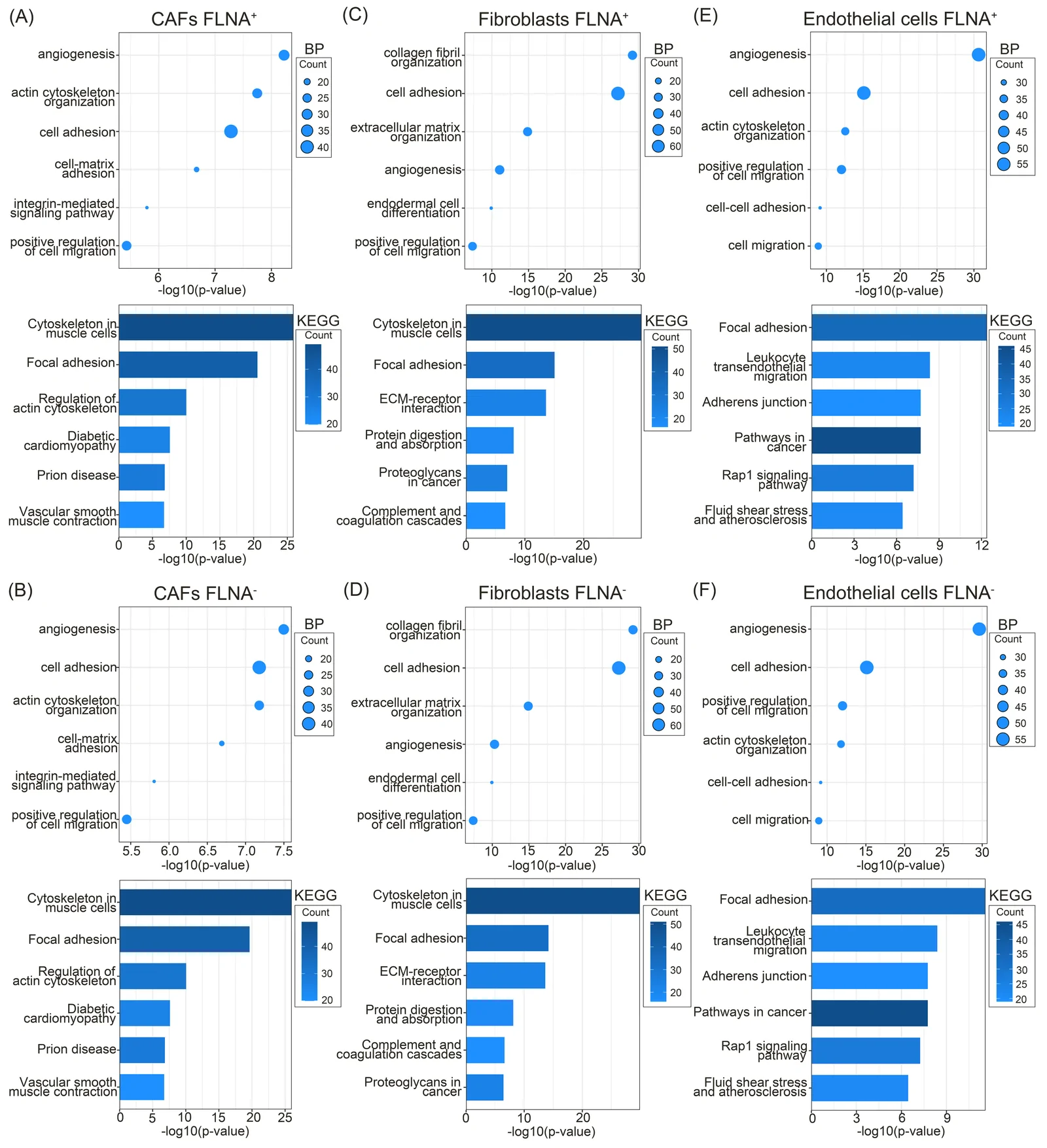
Biological process (BP) and pathway activation in cancer-associated fibroblasts (CAFs), fibroblasts, and endothelial cells from the GSE144735 single-cell dataset. (**A** and **B**) BP and Kyoto Encyclopedia of Genes and Genomes (KEGG) analyses comparing CAFs with high versus low FLNA expression. (**C** and **D**) BP and KEGG analyses of fibroblasts with high versus low FLNA expression. (**E** and **F**) BP and KEGG analyses of endothelial cells with high versus low FLNA expression.

**Figure 11:**
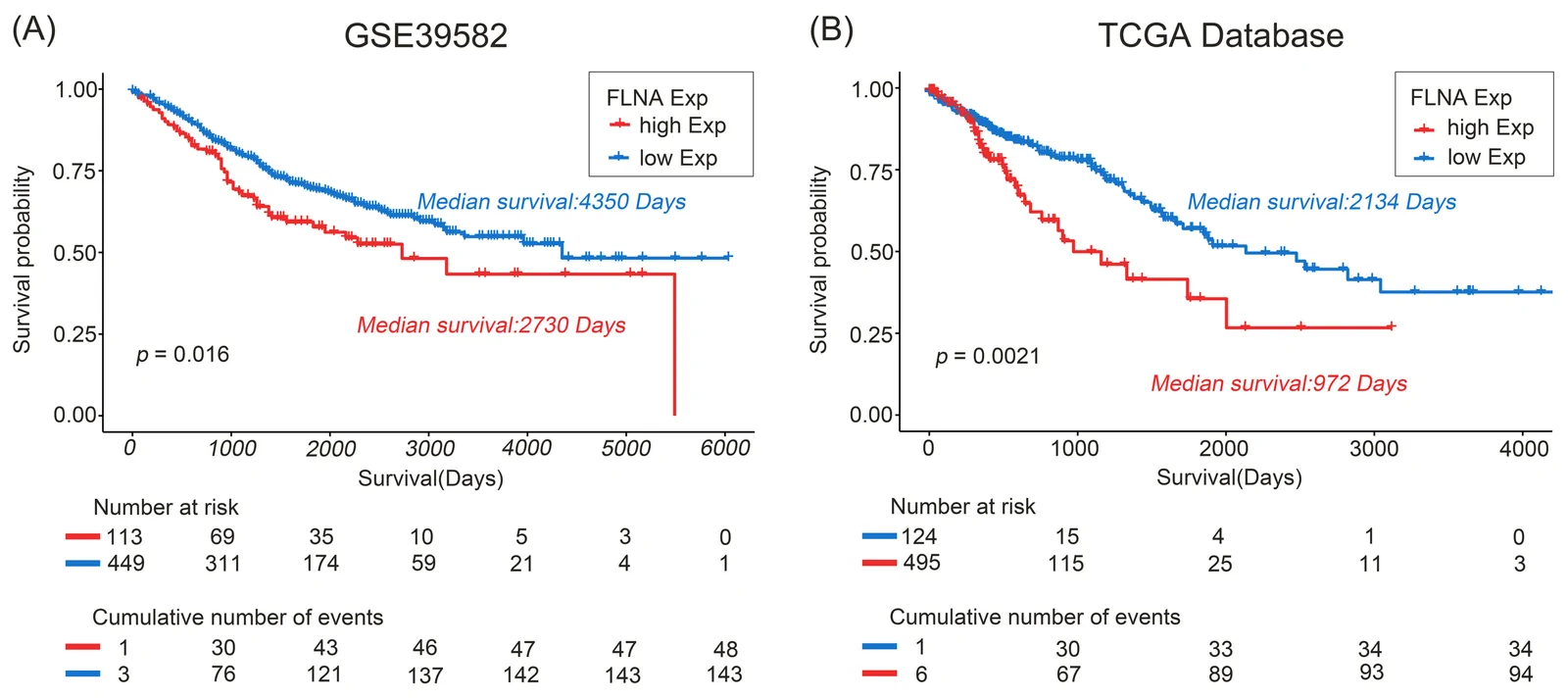
Prognostic significance of FLNA expression. Kaplan-Meier analysis with log-rank test for overall survival (OS) in the (**A**) GSE39582 training cohort and (**B**) The Cancer Genome Atlas (TCGA) validation cohort. The optimal cutoff (9.37, top 20.11%) determined in GSE39582 was converted to a standardized top 20% threshold for clinical applicability in the TCGA cohort.

**Table 1:** Univariable and multivariable analysis of prognostic factors for overall survival (OS) in the GSE39582 cohort

	Univariate analysis		Multivariate analysis	
Variable	HR (95%CI)	*p*-value	HR (95%CI)	*p*-value
FLNA expression	1.464 (1.055-2.031)	0.023	1.391 (1.001-1.933)	0.049
Age	1.024 (1.012-1.036)	9.24e-5	1.030 (1.018-1.042)	8.95e-07
M	4.726 (3.315-6.738)	9.38e-18	6.901 (0.952-50.027)	0.056
Stage		2.93e-15		0.405
Stage1 vs Stage0	0.218 (0.025-1.876)	0.165	0.274 (0.032-2.365)	0.239
Stage2 vs Stage0	0.390 (0.054-2.818)	0.350	0.444 (0.061-3.216)	0.421
Stage3 vs Stage0	0.479 (0.066-3.469)	0.467	0.556 (0.077-4.039)	0.562
Stage4 vs Stage0	1.941 (0.266-14.139)	0.513	0.380 (0.023-6.346)	0.501

Abbreviations: CI, confidence interval; HR, hazard ratio.

**Table 2:** Univariable and multivariable analysis of prognostic factors for overall survival (OS) in The Cancer Genome Atlas (TCGA) database

	Univariate analysis		Multivariate analysis	
Variable	HR (95%CI)	*p*-value	HR (95%CI)	*p*-value
FLNA expression	1.875 (1.279-2.747)	0.001	1.558 (1.056-2.299)	0.025
Age	1.031 (1.015-1.049)	2.36e-4	1.042 (1.025-1.059)	9.95e-07
M	3.652 (2.492-5.353)	3.09e-11	805.552 (3.79e-17-1.71e+22)	0.768
Stage		8.61e-10		0.007
Stage1 vs Stage0	82.458 (1.11e-31-6.14e+34)	0.909	39.537 (4.68e-35-3.34e+37)	0.931
Stage2 vs Stage0	131.924 (1.78e-31-9.79e+34)	0.899	61.516 (7.30e-35-5.19e+37)	0.922
Stage3 vs Stage0	247.295 (3.33e-31-1.84e+35)	0.887	127.605 (1.51e-34-1.08e+38)	0.909
Stage4 vs Stage0	565.514 (7.62e-31-4.20e+35)	0.870	0.381 (6.13e-42-2.37e+40)	0.984

Abbreviations: CI, confidence interval; HR, hazard ratio.

## Data Availability

All datasets analyzed in this study are publicly available from the following repositories: The transcriptomic data for the GSE39582 cohort (n = 566) and the GSE75316 cohort (n = 59) are available from the Gene Expression Omnibus (GEO) at (https://www.ncbi.nlm.nih.gov/geo/query/acc.cgi?acc=GSE39582) and (https://www.ncbi.nlm.nih.gov/geo/query/acc.cgi?acc=GSE75316), respectively. The single-cell RNA-sequencing data (GSE144735) are available from GEO at (https://www.ncbi.nlm.nih.gov/geo/query/acc.cgi?acc=GSE144735). The bulk RNA-sequencing and corresponding clinical data for The Cancer Genome Atlas (TCGA) colorectal cancer cohort (n = 633) can be accessed via the Genomic Data Commons (GDC) portal at (https://portal.gdc.cancer.gov/).
