# Supplementary material for: FLNA Expression in Cancer-Associated Fibroblasts Is Associated with Immunosuppression and Tumor Progression in Colorectal Cancer
Source: J Cancer. 2026 Oct 16;17(11):1879–94. doi: 10.7150/jca.134223 (PMC13686791; doi:10.7150/jca.134223)
Supplement: Supplementary file 1 [file jcav17p1879s1.pdf]

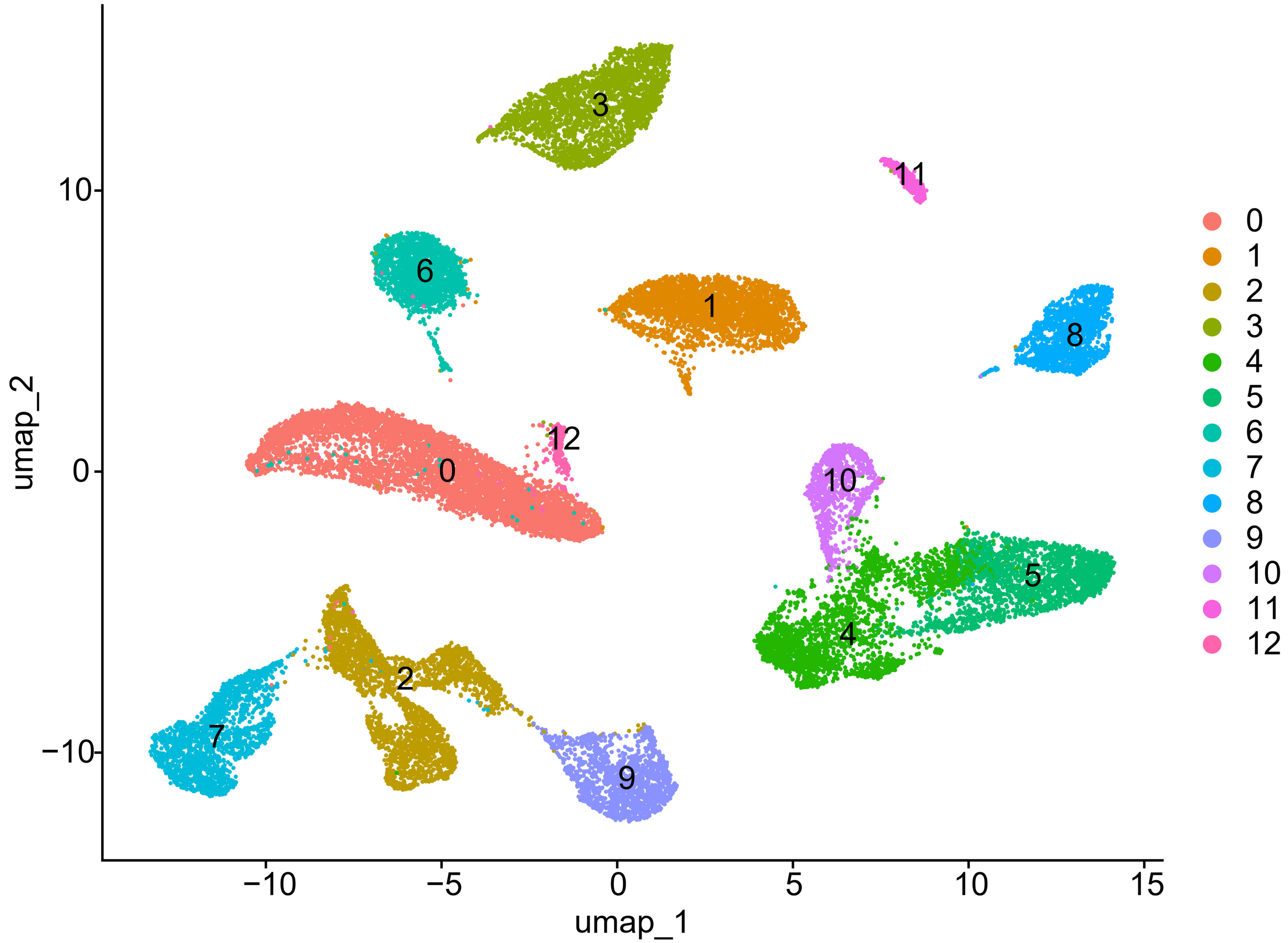

Supplementary Figure S1 Expression of FLNA across cell types in the colorectal cancer (CRC) tumor microenvironment (TME). (A) UMAP plot showing cell clusters based on transcriptomic profiles from the GSE144735 dataset.

Supplementary Table S1 Collinearity diagnostics (Variance Inflation Factor, VIF) for clinicopathological variables in the GSE39582 cohorts prior to multivariable Cox regression analysis.

Coefficients[a]

Model				Standardized Coefficients	t	Sig.	95.0% Confidence Interval for B		Collinearity Statistics	
				Beta			Lower Bound	Upper Bound	Tolerance	VIF
1	(Constant)	84.017	12.830		6.549	0.000	58.814	109.220		
	age	-0.492	0.120	-0.170	-4.110	0.000	-0.727	-0.257	0.979	1.022
	T	-0.154	2.919	-0.003	-0.053	0.958	-5.888	5.580	0.672	1.488
	N	-13.645	6.963	-0.174	-1.960	0.051	-27.324	0.034	0.211	4.749
	M	-42.555	8.749	-0.346	-4.864	0.000	-59.742	-25.368	0.330	3.032
	stage	7.319	5.993	0.149	1.221	0.223	-4.453	19.091	0.112	8.966
	FLNA	-2.521	4.051	-0.026	-0.622	0.534	-10.479	5.436	0.977	1.023

a. Dependent Variable: OS

Coefficients[a]

Model				Standardized Coefficients	t	Sig.	95.0% Confidence Interval for B		Collinearity Statistics	
				Beta			Lower Bound	Upper Bound	Tolerance	VIF
1	(Constant)	84.990	12.440		6.832	0.000	60.554	109.425		
	age	-0.503	0.118	-0.174	-4.278	0.000	-0.734	-0.272	0.978	1.022
	N	-12.373	6.135	-0.160	-2.017	0.044	-24.425	-0.322	0.257	3.898
	M	-40.701	8.022	-0.329	-5.074	0.000	-56.457	-24.945	0.383	2.609
	stage	6.467	4.960	0.132	1.304	0.193	-3.276	16.211	0.157	6.354
	FLNA	-3.565	3.897	-0.037	-0.915	0.361	-11.220	4.089	0.978	1.022

a. Dependent Variable: OS

Coefficients[a]

Model				Standardized Coefficients	t	Sig.	95.0% Confidence Interval for B		Collinearity Statistics	
				Beta			Lower Bound	Upper Bound	Tolerance	VIF
1	(Constant)	99.576	10.149		9.811	0.000	79.640	119.511		
	age	-0.499	0.118	-0.172	-4.236	0.000	-0.731	-0.268	0.978	1.022
	M	-31.410	6.585	-0.254	-4.770	0.000	-44.345	-18.476	0.572	1.749
	stage	-2.043	2.614	-0.042	-0.782	0.435	-7.178	3.091	0.570	1.755
	FLNA	-3.822	3.906	-0.040	-0.979	0.328	-11.494	3.849	0.979	1.021

a. Dependent Variable: OS

Supplementary Table S1. Collinearity diagnostics (Variance Inflation Factor, VIF) for clinicopathological variables in the TCGA cohorts prior to multivariable Cox regression analysis.

Coefficients[a]

Model				Standardized Coefficients Beta	t	Sig.	95.0% Confidence Interval for B Lower Bound	Upper Bound	Collinearity Statistics Tolerance	VIF
1	(Constant)	516.616	208.529		2.477	0.014	107.091	926.142		
	age	-1.391	2.285	-0.025	-0.608	0.543	-5.879	3.098	0.966	1.035
	T	-49.574	66.879	-0.044	-0.741	0.459	-180.917	81.770	0.448	2.232
	N	-295.125	127.149	-0.204	-2.321	0.021	-544.830	-45.419	0.209	4.792
	M	-369.525	139.325	-0.182	-2.652	0.008	-643.144	-95.907	0.343	2.912
	stage	221.272	101.103	0.293	2.189	0.029	22.718	419.826	0.090	11.136
	FLNA	-116.061	69.967	-0.068	-1.659	0.098	-253.467	21.345	0.949	1.054

a. Dependent Variable: OS

Coefficients[a]

Model				Standardized Coefficients Beta	t	Sig.	95.0% Confidence Interval for B Lower Bound	Upper Bound	Collinearity Statistics Tolerance	VIF
1	(Constant)	480.332	202.627		2.371	0.018	82.399	878.265		
	age	-1.498	2.280	-0.027	-0.657	0.512	-5.975	2.980	0.970	1.031
	N	-249.844	111.472	-0.173	-2.241	0.025	-468.761	-30.927	0.271	3.686
	M	-323.969	124.991	-0.159	-2.592	0.010	-569.434	-78.503	0.426	2.346
	stage	170.304	74.092	0.226	2.299	0.022	24.796	315.812	0.167	5.985
	FLNA	-124.170	69.080	-0.073	-1.797	0.073	-259.835	11.496	0.973	1.028

a. Dependent Variable: OS

Coefficients[a]

Model				Standardized Coefficients Beta	t	Sig.	95.0% Confidence Interval for B Lower Bound	Upper Bound	Collinearity Statistics Tolerance	VIF
1	(Constant)	661.273	186.463		3.546	0.000	295.085	1027.461		
	age	-1.133	2.282	-0.020	-0.497	0.620	-5.614	3.348	0.975	1.025
	M	-203.021	113.113	-0.100	-1.795	0.073	-425.160	19.119	0.524	1.909
	stage	33.764	42.311	0.045	0.798	0.425	-49.329	116.858	0.516	1.939
	FLNA	-126.171	69.302	-0.074	-1.821	0.069	-262.272	9.930	0.973	1.028

a. Dependent Variable: OS
